# Impaired H-Reflex Adaptations Following Slope Walking in Individuals With Post-stroke Hemiparesis

**DOI:** 10.3389/fphys.2019.01232

**Published:** 2019-10-01

**Authors:** Jing Nong Liang, Yun-Ju Lee, Eric Akoopie, Brooke Conway Kleven, Trisha Koch, Kai-Yu Ho

**Affiliations:** ^1^Department of Physical Therapy, University of Nevada, Las Vegas, Las Vegas, NV, United States; ^2^Department of Industrial Engineering and Engineering Management, National Tsing Hua University, Hsinchu, Taiwan

**Keywords:** locomotor control, H-reflex, post-stroke hemiparesis, slope walking, spinal cord plasticity

## Abstract

**Background and Purpose:**

Short term adaptations in the Ia afferent-motoneuron pathway, as measured using the H-reflex, in response to altered ground reaction forces (GRFs) applied at the feet during slope walking have been observed in the non-impaired nervous system. The ability of the stroke-impaired nervous system to adapt to altered GRFs have not been examined. The purpose of this study was to examine the acute effects of altered propulsive and braking forces applied at the feet, which naturally occurs when walking on different slopes, on adaptations of the H-reflex pathway in individuals with chronic post-stroke hemiparesis.

**Methods:**

Twelve individuals chronically post-stroke and 10 age-similar non-neurologically impaired controls walked on an instrumented treadmill for 20 min under level, upslope and downslope conditions. GRFs were measured during walking and soleus H-reflexes were recorded prior to and immediately after walking. A 3 (limbs: paretic, non-paretic, and non-impaired) × 3 (slope: level, upslope, downslope) mixed factorial ANOVA was conducted on the propulsive and braking forces. A 2 (limb: paretic and non-impaired) × 2 (time: pre and post) × 3 (slope: level, upslope, and downslope) mixed factorial ANOVA was conducted to assess the soleus H-reflex amplitudes.

**Results:**

In both post-stroke and non-impaired groups, during downslope walking, peak propulsive forces decreased, while peak braking forces increased. In contrast, during upslope walking, peak propulsive forces increased and peak braking forces decreased. We observed reduced soleus H-reflex amplitudes immediately following 20 min of level, downslope and upslope walking in non-impaired individuals but not in the paretic legs of individuals with chronic post-stroke hemiparesis.

**Discussion and Conclusion:**

Similar pattern of change in peak propulsive and braking forces with respect to different slopes was observed in both individuals post-stroke and non-impaired individuals, but the magnitude of GRFs were smaller in individuals post-stroke due to the slower walking speed. Our results suggested that impaired modulation of the H-reflex pathway potentially underlies the lack of neuroadaptations in individuals with chronic post-stroke hemiparesis.

## Introduction

Stroke is the leading cause of long-term adult disabilities (Benjamin et al., 2018). While majority of stroke survivors regain independent ambulation, there often remains impaired motor control, and thus limitations in walking, which can present as a safety concern when participating in daily physical activities (Olney and Richards, 1996).

To execute successful locomotion, leg muscles have to be coordinated so as to generate foot forces of appropriate magnitude and direction to propel the center of mass forward (Neptune et al., 2001). In individuals with chronic post-stroke hemiparesis, however, inappropriate direction of foot forces during locomotion has been reported (Bowden et al., 2006; Turns et al., 2007; Liang and Brown, 2013, 2014), specifically, reduced propulsive forces accompanied by exaggerated braking forces have been observed in paretic legs (Olney and Richards, 1996; Bowden et al., 2006; Turns et al., 2007). Furthermore, the impaired paretic foot force control capabilities during locomotion are exacerbated when the stroke-impaired nervous system is required to control for postures in addition to the locomotor task. During non-postural loaded locomotion, foot force directional control was well regulated in individuals post-stroke, but excessive forward directed shear forces were generated during postural loaded locomotion, and further exaggerated with higher postural loads (Liang and Brown, 2013, 2014). It has also been observed that impaired H-reflex gain was associated with this defective interaction of postural and locomotor control in individuals post-stroke (Liang and Brown, 2015). Functionally, insufficient horizontal forces in the anterior-posterior direction contribute to reduced propulsion (Bowden et al., 2006), thus associated with slow walking speeds, and excessive horizontal forces on low friction surfaces could lead to slips and falls (Redfern et al., 2001).

Sloped surfaces are routinely encountered in daily activities, specifically where there is a transition between surfaces of different elevation, such as ramps. Introduction of sloped surfaces, both incline and decline, is challenging and present increased potential for loss of balance and falls compared to level surfaces (Sheehan and Gottschall, 2012), especially for individuals with post-stroke hemiparesis.

While the short term H-reflex adaptations in response to walking under altered propulsive and braking force conditions, via walking on inclined or declined slopes, have been examined in non-impaired individuals (Sabatier et al., 2015; Arnold et al., 2017), little is known about the H-reflex adaptations following walking on inclined/declined slopes in the stroke-impaired nervous system. The H-reflex is a commonly used electrophysiological test to quantify excitatory behavior of the monosynaptic group Ia afferent volleys in the spinal cord circuitry (Schieppati, 1987). In the non-impaired nervous system, the spinal circuitry undergoes acute adaptations in response to a task or exercise. Soleus (SOL) H-reflex depressions have been observed following single sessions of level walking (Sabatier et al., 2015), downslope walking (Sabatier et al., 2015), downslope running (Bulbulian and Bowles, 1992), and active leg cycling (Motl and Dishman, 2003; Motl et al., 2003). Increased presynaptic inhibition resulting from the repetitive stretch-shortening of the targeted muscle from these cyclical tasks, reciprocal inhibition from repetitive activation of antagonist muscle, or prolonged post-activation depression have been suggested as possible underlying mechanisms (Hultborn et al., 1996; Motl et al., 2003). In the stroke-impaired nervous system, the reduction in SOL H-reflexes has been observed after active pedaling (Tanuma et al., 2017). While this has implications for therapeutic interventions in people post-stroke, since the size of the H-reflex has been associated with spasticity in the neurologically impaired (Levin and Hui-Chan, 1993), it also suggests that the impaired Ia afferent pathway potentially has capabilities to adapt in response to external perturbations. However, active cycling employed by the earlier study is a seated task requiring minimal control for postural loads. Potential for adaptation in the stroke-impaired nervous system has not been examined in locomotor tasks involving postural control.

The primary purpose of this study was to examine the immediate effects of altered propulsive and braking forces applied at the feet, which naturally occurs when walking on different slopes, on adaptations of the Ia afferent pathway, as measured using the H-reflex, in individuals with chronic post-stroke hemiparesis. We hypothesized that the ability to modulate H-reflex excitability in response to altered propulsive and braking forces would be reduced in the post-stroke nervous system.

## Materials and Methods

### Participants

Twelve individuals (4 females, 8 males; age = 70.54 ± 9.76 years), who had sustained a single, unilateral cortical or subcortical stroke, more than 6 months postictus (7.39 ± 8.41 years) prior to the study, and had residual lower limb hemiparesis, participated in this study. Ten age-similar non-neurologically impaired individuals (6 females, 4 males; age 59.36 ± 11.45 years) were recruited as controls.

Individuals post-stroke were recruited only if they were able to walk on a treadmill independently without assistive devices (cane/quadcane/ankle-foot orthosis). Participants were excluded if they had other neurological conditions, expressive or receptive aphasia, severe concurrent medical problems such as severe cardiac disease, history of poorly controlled brittle diabetes, active cancer, orthopedic conditions affecting the legs, history of hip or knee replacement, peripheral nerve injury in the lower limb, or the inability to comprehend verbal instructions. Each participant received written and verbal information about the experiment procedures before giving written consent. All participants signed the informed consent before participation. The protocol was approved by the Institutional Review Board at the University of Nevada, Las Vegas.

### Procedures

Each participant was tested over 2 sessions separated by at least 7 days. For each session, each participant either walked on the level and the upslope, or the level and the downslope conditions, with at least 2 h in between the conditions. The order of upslope and downslope conditions was determined in an alternate order, such that half the participants walked level and upslope conditions during their first session, and the remainder walked level and downslope conditions during their first session. In the first session, we recorded each participant’s body weight and quantified the lower extremity functional motor level using the Fugl-Meyer Assessment of motor function (Fugl-Meyer et al., 1975).

### SOL H-Reflex Elicitation and Electromyography (EMG) Recordings

The H-reflexes and M waves were recorded before and immediately after each 20-min walking session. In individuals with chronic post-stroke hemiparesis, H-reflexes were elicited from the paretic SOL muscle and in non-impaired individuals, the right SOL muscle was tested. To elicit SOL H-reflexes and M-waves, bipolar self-adhesive Ag-AgCl electrodes (2.2 × 2.2 cm for the cathode and 2.2 × 3.5 cm for the anode, VerMed, Inc., Bellows Falls, VT, United States) were placed over the popliteal fossa to stimulate the tibial nerve, using a constant current stimulator and isolation unit (DS7A, Digitimer Ltd., Welwyn Garden City, United Kingdom), with a square pulse stimuli of 1ms duration, a current range of 50 μA∼200 mA, and total output capability of 400 V. The stimulating electrodes were placed at a spot where the H-reflex threshold was minimized and stimulation of other nerves avoided. To avoid between session variability in placement of electrodes, after the first session, the electrode positions were mapped relative to bony landmarks, scars, or moles on the skin. The same trained researcher placed the electrodes each session for all participants.

The H-reflexes and M-waves were measured while the participants maintained a natural standing posture with arms by their sides, and with stable levels of SOL background EMG activity. An additional electrode was placed over the belly of the tibialis anterior muscle (TA) to monitor antagonist EMG activity. Each electrical stimulus would occur only after the participant had maintained constant level of rectified SOL and TA EMG activity within a set window of mean background EMG during static standing ± 5% for at least 2 s, and with at least 8 s inter-stimulus interval to avoid effects of low frequency depression (Chang et al., 2013). Prior to and immediately after 20 min of walking, the intensity of the stimulator was increased in small increments until the maximal SOL H-reflex and subsequently the maximal M-wave was obtained, generating a recruitment curve. Then, 10 maximal SOL H-reflexes were recorded. For each participant, the 2 sessions were scheduled at same time of day to avoid diurnal variations in H-reflex sizes (Carp et al., 2006; Lagerquist et al., 2006). SOL H-reflexes and TA EMG activity were amplified, band-pass filtered (3–3000 Hz), sampled at 3200 Hz, and stored for offline analysis.

### Slope Walking

Participants wore a safety harness attached to an overhead support, and walked on a dual belt instrumented treadmill (Fully Instrumented Treadmill, Bertec Corp., Columbus, OH, United States) at a sample frequency of 2000 Hz. The safety harness only served as a safety mechanism in case of a fall and did not support any body weight during walking. Each participant walked for 20 min under each of the 3 slope conditions: a level walking condition with treadmill at 0° incline/decline, an upslope condition with treadmill at 5° incline, and a downslope condition with treadmill at 5° decline. This relatively small incline angle was selected to ensure that individuals post-stroke were able to complete 20 min of continuous walking. This duration was selected because previous studies reported that 20 min of walking was sufficient to induce a change in H-reflex amplitude (Sabatier et al., 2015; Arnold et al., 2017). We first determined the walking speed across the 3 conditions by asking participants to walk with their comfortable speeds for level walking on the instrumented treadmill. The speed was recorded and used for upslope and downslope conditions. All self-selected walking speeds were determined within two 30-s trials. During the 20 min of walking for each condition, should the participant need to slow down, we would reduce the speed of the treadmill to accommodate, and the average walking speed during the 20-min walking session was reported. Heart rate and blood pressure were monitored prior to, during and after walking. GRFs were recorded every other minute for 60 s, for a total of 10 trials per walking condition for each participant.

### Data Processing

All forces and reflex data were processed and analyzed offline using custom MATLAB scripts (Mathworks, Natick, MA, United States). The peak-to-peak amplitudes of the maximal SOL H-reflexes and the maximal M-waves were calculated and averaged. GRFs were filtered with a 20 Hz low-pass, 2nd order, zero-lag Butterworth filter. The vertical component of the GRF was used to determine the stance phase of gait cycle. The time point where the vertical GRF exceeded zero newton and remained for a continuous period of at least 50 ms was denoted as heel contact. Subsequently, the time point where the vertical GRF reached zero newton and remained for a continuous period of at least 50 ms was denoted as toe off. The period from heel contact to toe off was considered the stance phase. The peak propulsive and braking forces during the stance phase were defined as highest positive and negative horizontal GRF in the anterior-posterior detected by the force plates, respectively. For each 60 s trial, the first and last five stance phases were removed from analysis. The mean propulsive and braking forces were averaged over the remaining stance phases, and normalized to the individual’s body weight. The same procedure was repeated for GRF data from the other force platform.

Data from the level walking condition during the first session was used in the analysis for comparison with upslope and downslope conditions. Level walking condition from the second session was used for control purposes, to ensure that we were able to obtain consistent recordings across sessions.

### Statistical Analysis

Data were tested for homogeneity and sphericity, and it was confirmed that no assumptions were violated.

A 2 (limb: paretic and non-impaired) × 2 (time: pre and post) × 3 (slope: level, upslope, and downslope) mixed factorial ANOVA was conducted on the H_max_/M_max_. Any significant interacting factors were broken down using two-way ANOVAs and *post hoc* simple main effects as described below. Significant main effects were reported if there were no significant interactions.

A 3 (limb: paretic, non-paretic, and non-impaired) × 3 (slope: level, upslope, downslope) mixed factorial ANOVA was conducted on the propulsive and braking forces. When there was a significant interaction effect, we further examined simple main effects using a repeated measures ANOVA with a Bonferroni correction for each limb. Significant main effects were reported if there were no significant interactions. *P*-values less than or equal to 0.05 were considered statistically significant.

## Results

**FIGURE 1:**
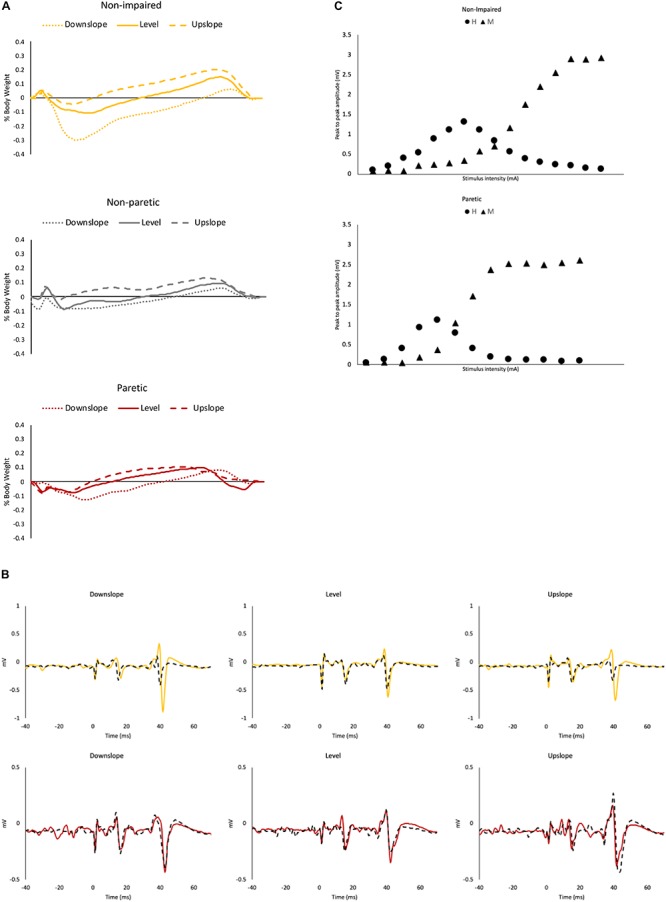
Representative data. **(A)** Ground reaction force data from a representative non-impaired leg (yellow), non-paretic leg (gray) and paretic leg (red), under downslope (dotted), level (solid) and upslope (dashed) conditions. **(B)** Soleus maximal H-reflexes with control M waves from a representative non-impaired (yellow) and a paretic leg (red) pre (solid) and post (dashed) walking under downslope, level and upslope conditions. **(C)** Representative recruitment curve from a non-impaired leg and a paretic leg. Each data point represents a mean of 3 values.

Participant characteristics are presented in Table 1. Representative propulsive forces, braking forces, SOL H-reflexes with control M waves pre/post walking under 3 slope conditions, and recruitment curves are presented in Figure 1. When comparing the self-selected walking speeds for each condition using independent *t*-tests, non-impaired individuals walked at a faster speed (Level = 1.07 ± 0.25 m/s; Upslope = 1.00 ± 0.22 m/s; Downslope = 1.04 ± 0.21 m/s) than individuals post-stroke (Level = 0.63 ± 0.25 m/s; Upslope = 0.57 ± 0.19 m/s; Downslope = 0.60 ± 0.24 m/s) for all 3 conditions (*p* < 0.05). Walking speeds decreased when comparing upslope with level walking in both groups (*p* < 0.05), but no difference when comparing downslope with level walking. Despite the slower walking speed for upslope walking, heart rate was higher following upslope compared to downslope and level, for both groups (Table 1). For each participant, we successfully obtained comparable M_max_ amplitudes for both sessions (ICC = 0.95, 95% CI), supporting the validity to compare the H_max_/M_max_ ratios recorded. We did not observe a statistically significant difference when comparing the H_max_/M_max_ ratios between paretic (0.40 ± 0.07) and non-impaired legs (0.37 ± 0.06) using independent *t*-tests for pre-walking conditions (*p* > 0.05).

**TABLE 1:** Characteristics of participants.

**Variable**	**Non-impaired (*n* = 10)**	**Stroke-impaired (*n* = 12)**	***p***
Age (years) (mean ± SD)	59.36 ± 11.45	70.54 ± 9.76	0.03^a^
Sex (female/male)	6/4	4/8	
Time since stroke onset (years) (mean ± SD)		7.39 ± 8.41	
Paretic side (left/right)		10/2	
F-M score (LE)^b^ (mean ± SD)		28.42 ± 2.15	
Self-selected walking speed(m/s) (mean ± SD)			
Level	1.07 ± 0.25	0.63 ± 0.25	0.00^a^
Upslope	1.00 ± 0.22^c^	0.57 ± 0.19^c^	0.00^a^
Downslope	1.04 ± 0.21	0.60 ± 0.24	0.00^a^
Heart rate (bpm) (mean ± SD)			
Level (Pre/Post)	72.89 ± 7.88 / 79.00 ± 8.77	75.33 ± 9.04 / 81.17 ± 10.63	
Upslope	72.67 ± 8.89 / 91.67 ± 9.27^d^	75.75 ± 9.07 / 89.33 ± 10.56^d^	0.00^d^
Downslope	73.11 ± 6.64 / 79.67 ± 8.03	74.00 ± 7.77 / 79.83 ± 10.50	
Baseline H_max_/M_max_	0.37 ± 0.06	0.40 ± 0.07	>0.05^a^

*^*a*^Evaluated by means of independent *t*-test. ^*b*^Fugl-Meyer Assessment - Lower Extremity motor function (total = 34). ^*c*^Statistically significant difference compared to level walking, evaluated by paired *t*-test. ^*d*^Statistically significant difference compared to pre walking, evaluated by paired *t*-test.*

### H_max_/M_max_

**FIGURE 2:**
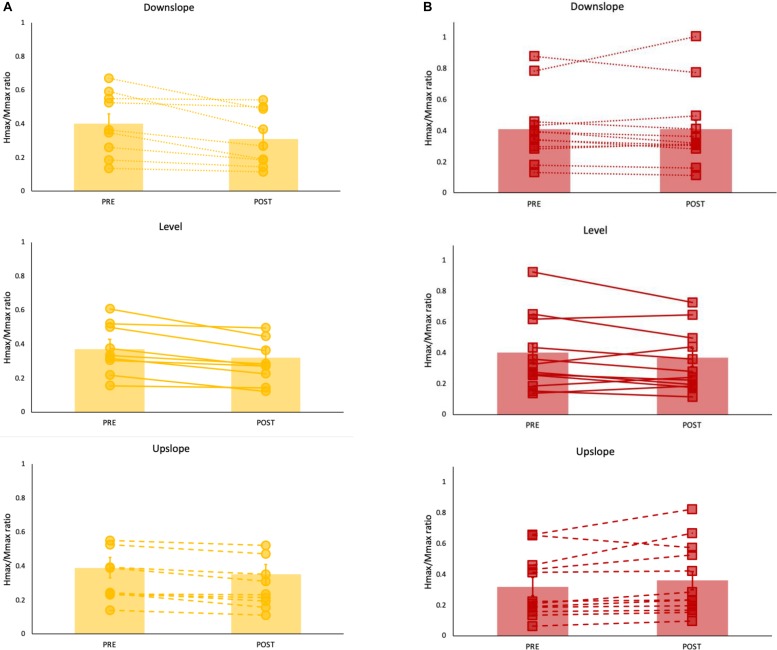
Hmax/Mmax ratios (mean ± SE) (bar graph) with individual data superimposed for each **(A)** non-impaired (yellow) and **(B)** paretic (red) leg pre and post walking under level (solid line), upslope (dashed) and downslope (dotted) conditions. Each data point represents a mean of 10 values.

**FIGURE 3:**
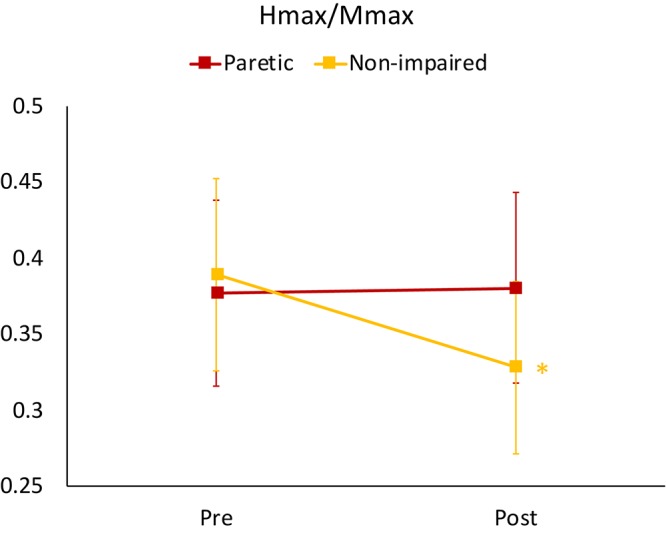
Mean H_max_/M_max_ ratios for non-impaired and paretic limbs across all three walking conditions (level, upslope, and downslope) measured prior to (Pre) and immediately after (Post) 20 min of walking. ^∗^indicates a significant difference from pre walking (*p* < 0.00).

For the H_max_/M_max_, the 3-way ANOVA revealed no significant interaction between limb, slope and time. There was a statistically significant interaction between limb and time [*F*(1,19) = 16.84, *p* = 0.001], suggesting that the change in H_max_/M_max_ after walking was different for stroke-impaired compared to non-impaired group. Simple effects analysis showed that in paretic legs, on average across all slope conditions, post walking H_max_/M_max_ (0.38 ± 0.06) was not different when compared to pre walking H_max_/M_max_ (0.38 ± 0.06) (*p* = 0.766). In non-impaired legs, however, on average across all slope conditions, H_max_/M_max_ was reduced after walking (0.33 ± 0.06), compared with pre walking H_max_/M_max_ (0.39 ± 0.06) (*p* = 0.00) (Figures 2, 3 and Table 2).

**TABLE 2:** H_max_/M_max_ ratios for non-imapried limb and paretic limb for each walking condition.

**Limb**	**Non-impaired *n* = 10**		**Paretic *n* = 12**	
**Time**	**Pre**	**Post**	**Pre**	**Post**
Level	0.37 ± 0.06	0.32 ± 0.05	0.40 ± 0.07	0.37 ± 0.06
Downslope	0.40 ± 0.06	0.31 ± 0.06	0.41 ± 0.06	0.41 ± 0.07
Upslope	0.39 ± 0.06	0.35 ± 0.08	0.32 ± 0.06	0.36 ± 0.07
Average	0.39 ± 0.06	0.33 ± 0.06^∗^	0.38 ± 0.06	0.38 ± 0.06

*^∗^Denotes statistically significant difference from pre walking conditions (*p* < 0.00).*

### Propulsive and Braking Forces

**FIGURE 4:**
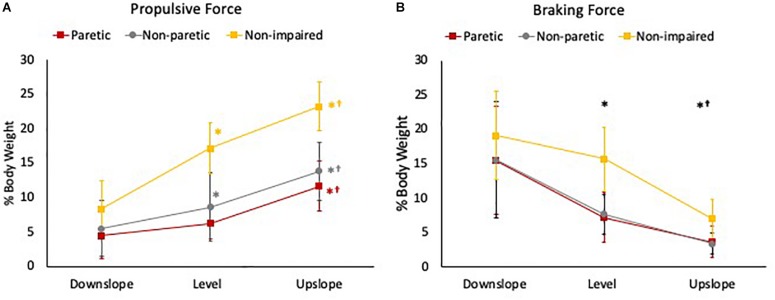
**(A)** Peak propulsive force and **(B)** peak braking force during the stance phase of gait of the 3 legs (paretic limb, non-paretic limb, and non-impaired limb) during downslope, level, and upslope walking. The error bars indicate the standard errors. ^∗^Indicates a significant difference from downslope walking and **^†^**indicates a significant difference from level walking (*p* < 0.00). Red, gray, and yellow symbols highlight the differences in the paretic, non-paretic, and non-impaired limbs, respectively. Black symbols highlight the differences across all 3 limbs.

For peak propulsive forces, there was a statistically significant interaction between slope and limb [*F*(2.83, 43.88) = 10.14, *p* < 0.001]. Simple main effect analysis showed that in non-impaired and non-paretic legs, peak propulsive forces were greater in the upslope (non-impaired = 23.20 ± 3.5%BW; non-paretic = 13.79 ± 4.20%BW) compared to level (non-impaired = 17.15 ± 3.65%BW; non-paretic = 8.63 ± 5.01%BW) and greater during level compared to the downslope (non-impaired = 8.35 ± 4.03%BW; non-paretic = 5.46 ± 4.04%BW) condition (*p* < 0.001). In the paretic legs, peak propulsive forces were greater only when walking upslope (11.67 ± 3.61%BW) (*p* < 0.001) compared to level (6.25 ± 2.30%BW) and downslope (4.42 ± 3.38%BW) conditions (Figure 4A).

For peak braking forces, there was no statistically significant interaction between slope and limb [*F*(2.29, 35.46) = 1.63, *p* = 0.21]. There was a statistically significant main effect of slope [*F*(1.14, 35.46) = 73.77, *p* < 0.001]. *Post hoc* analysis showed that the overall mean peak braking forces across the 3 limbs were significantly greater when walking downslope (16.60 ± 7.65%BW) compared to level (9.85 ± 5.27%BW) and upslope (4.54 ± 2.73%BW), and greater during level compared to upslope conditions (*p* < 0.001) (Figure 4B).

## Discussion

The aim of this study was to examine the acute effects of altered propulsive and braking GRFs, using upslope, level and downslope walking, on H-reflex excitability in individuals with chronic post-stroke hemiparesis. Our main findings were that immediately following 20 min of walking, regardless of the slope conditions, we observed reduced SOL H-reflex amplitudes in non-impaired legs but not in paretic legs of individuals post-stroke, supporting our hypothesis.

In non-impaired individuals, during downslope walking, peak propulsive forces decreased while peak braking forces increased significantly. In contrast, during upslope walking, peak propulsive forces increased and peak braking forces decreased. These observations are in line with data reported in the literature (Lay et al., 2006), thus allowing us to investigate the effects of altered magnitudes of anterior-posterior GRFs applied at the feet on H-reflex adaptations. In individuals post-stroke, we observed a similar pattern of change in both propulsive and braking forces with respect to different slopes. However, in terms of magnitude, we observed a smaller magnitude in propulsive forces generated by non-paretic and paretic limbs, compared to the non-impaired. This difference in magnitude is more likely attributable to the difference in walking speed, where the non-impaired participants walked at a faster self-selected walking speed than individuals post-stroke, and less likely attributable to the difference in age between the two groups. Positive relationship between GRFs and walking speed has been documented (Peterson et al., 2011). Previous study reported similar magnitudes of GRFs during slope walking in older adults compared to the relatively younger non-impaired individuals in our study (Franz and Kram, 2013).

To minimize confounding effects associated with exercise intensity and muscle fatigue, we monitored the heart rate during walking to ensure that a submaximal intensity of exercise was set for each condition. Both stroke-impaired and non-impaired groups walked at slower walking speeds during upslope versus level conditions. This was in accordance with previous studies where walking speeds decrease with inclination of the treadmill during the upslope condition (Kawamura et al., 1991; Sun et al., 1996). Despite the greater heart rate following upslope walking, the self-selected walking speeds were also slower than level and downslope for both groups. This was in agreement with previous literature reporting lower metabolic cost associated with eccentric exercises (Minetti et al., 2002), in this case, downslope walking.

In the non-impaired individuals, excitability of the Ia afferent pathway, as assessed by the SOL H_max_/M_max_ ratio was reduced immediately after walking, regardless of slope type. This reduced excitability of H-reflex pathway we observed immediately after slope walking could be explained by the effects of post-activation depression, which is when preceding increased activation of the Ia afferents results in a transmitter depletion, thus resulting in long-lasting depression of transmission across the motoneuron synapse (Hultborn et al., 1996). Similar observations of short-term reductions in H-reflex amplitudes have been previously reported following single bouts of level (Thompson et al., 2006; Sabatier et al., 2015) and downslope (Sabatier et al., 2015; Arnold et al., 2017) treadmill walking, running (Bulbulian and Bowles, 1992), as well as other locomotor tasks, such as active and passive cycling (Motl et al., 2003).

In contrast, regardless of the slope condition, we did not observe any changes in paretic SOL H_max_/M_max_ following 20 min of walking. Following non-postural loaded locomotor tasks such as active pedaling, reduction in paretic SOL H-reflexes have been observed after 30 min, compared to pre-pedaling conditions (Tanuma et al., 2017). Impaired post-activation depression or even facilitation, assessed as frequency related depression of the SOL H-reflex, has been previously reported in individuals with central nervous system lesions (Ishikawa et al., 1966; Schindler-Ivens and Shields, 2000; Masakado et al., 2005; Chang et al., 2013) as a result of neuromuscular adaptions. Prolonged robotic-assisted gait training of 4 weeks has been shown to partially restore the maladapted post-activation depression in individuals post-stroke (Trompetto et al., 2013). Thus, the intensity of slope walking in terms of duration or slope elevation, may not be sufficient to induce adaptations.

While we observed a higher mean H_max_/M_max_ ratio in the paretic legs compared to non-impaired, it did not reach statistical significance. Previously, conflicting results have been reported in the stroke-impaired nervous system; while some observed abnormally increased excitability (Thompson et al., 2009) in the paretic limbs, compared to the non-impaired, others reported no difference (Faist et al., 1994) due to the large variability in type and site of lesions. Furthermore, contrasting results also exist between different muscles examined (Aymard et al., 2000). Increased excitability of spinal reflexes has been associated with spasticity and hypertonia, and negatively impacts motor function, activity performance and functional status in neurologically impaired individuals (Pizzi et al., 2005; Zorowitz et al., 2013). Participants with post-stroke hemiparesis recruited in this study are skewed toward higher functional status, as suggested by the high lower extremity F-M scores. Clinically, restoration of increased H-reflex amplitudes can translate into reduced spasticity and improvements in functional ability in the neurologically impaired population (Manella and Field-Fote, 2013; Manella et al., 2013; Thompson et al., 2013). Thus, exploring paradigms that could induce this adaptation has significant clinical impact.

In the current study, because upslope walking is a metabolically challenging task for individuals post-stroke, in our attempt to match the elevation angles between upslope and downslope, we chose an incline/decline of 5 degrees, which is a smaller elevation than ramps that individuals encounter in the community. Future studies will examine incline/decline magnitudes comparable to ramps commonly encountered in the community on neural adaptations, as well as explore reflex adaptations during the slope walking task in the stroke-impaired nervous system. Additionally, walking speed was different in the non-impaired versus the stroke-impaired group, thus resulting in different magnitudes of GRFs in the 2 groups. Systematic exploration of various walking speeds and/or incline/decline angles would provide further insight.

## Conclusion

Despite the similar pattern of change in GRFs with respect to different slopes in both groups, we observed lack of acute slope-induced H-reflex adaptations in individuals with chronic post-stroke hemiparesis compared to non-neurologically impaired individuals. These observations provide early evidence for activity-dependent spinal plasticity in the non-impaired nervous system, and that the stroke-impaired nervous system may require repeated or prolonged exposures to reveal similar adaptations.

## Data Availability Statement

The datasets generated for this study are available on request to the corresponding author.

## Ethics Statement

This study was approved by the Institutional Review Board at the University of Nevada, Las Vegas.

## Author Contributions

JL conceived and designed the experiments, analyzed the data, and prepared the manuscript. Y-JL and K-YH contributed to the analysis and preparation of the manuscript. EA, BK, and TK collected the data.

## Conflict of Interest

The authors declare that the research was conducted in the absence of any commercial or financial relationships that could be construed as a potential conflict of interest.
